# Quantifying the effectiveness of climate change mitigation through forest plantations and carbon sequestration with an integrated land-use model

**DOI:** 10.1186/1750-0680-3-3

**Published:** 2008-04-15

**Authors:** Jelle G van Minnen, Bart J Strengers, Bas Eickhout, Rob J Swart, Rik Leemans

**Affiliations:** 1 Environmental Systems Analysis Group, Wageningen University, P.O. Box 47, NL-6700 AA Wageningen, The Netherlands; 2 Netherlands Environmental Assessment Agency (MNP), P.O. Box 303, NL-3720 AH Bilthoven, The Netherlands

## Abstract

**Background:**

Carbon plantations are introduced in climate change policy as an option to slow the build-up of atmospheric carbon dioxide (CO_2_) concentrations. Here we present a methodology to evaluate the potential effectiveness of carbon plantations. The methodology explicitly considers future long-term land-use change around the world and all relevant carbon (C) fluxes, including all natural fluxes. Both issues have generally been ignored in earlier studies.

**Results:**

Two different baseline scenarios up to 2100 indicate that uncertainties in future land-use change lead to a near 100% difference in estimates of carbon sequestration potentials. Moreover, social, economic and institutional barriers preventing carbon plantations in natural vegetation areas decrease the physical potential by 75–80% or more.

Nevertheless, carbon plantations can still considerably contribute to slowing the increase in the atmospheric CO_2 _concentration but only in the long term. The most conservative set of assumptions lowers the increase of the atmospheric CO_2 _concentration in 2100 by a 27 ppm and compensates for 5–7% of the total energy-related CO_2 _emissions. The net sequestration up to 2020 is limited, given the short-term increased need for agricultural land in most regions and the long period needed to compensate for emissions through the establishment of the plantations. The potential is highest in the tropics, despite projections that most of the agricultural expansion will be in these regions. Plantations in high latitudes as Northern Europe and Northern Russia should only be established if the objective to sequester carbon is combined with other activities.

**Conclusion:**

Carbon sequestration in plantations can play an important role in mitigating the build-up of atmospheric CO_2_. The actual magnitude depends on natural and management factors, social barriers, and the time frame considered. In addition, there are a number of ancillary benefits for local communities and the environment. Carbon plantations are, however, particularly effective in the long term. Furthermore, plantations do not offer the ultimate solution towards stabilizing CO_2 _concentrations but should be part of a broader package of options with clear energy emission reduction measures.

## Background

Climate on earth is changing and this has led to a series of impacts on the environment and human society [1]. This climate change is most likely caused by the increased greenhouse gas concentration with carbon dioxide (CO_2_) as the most important gas [2]. The United Nations Framework Convention on Climate Change (UNFCCC) in its mandate to limit future climate change and its impacts, aims to 'stabilize greenhouse gas (GHGs) concentrations in the atmosphere at a level that would prevent dangerous anthropogenic interference with the climate system' (Article 2 [3]). Many studies have compared emission reduction strategies to achieve different stabilization levels of CO_2 _and quantified their consequences (e.g. [4,5]). Most of these studies concentrate on reducing energy-related CO_2 _emissions and ignore abatement options that enhance CO_2 _uptake (or increase C sinks) by the biosphere. Such uptake also slows down the concentration increase.

The Kyoto Protocol, drafted in 1997 and entered into force in 2005, includes quantitative targets for industrial countries (the so-called "Annex B") to limit the emissions of six GHGs (CO_2_, CH_4_, N_2_O, and three fluorinated gases) by the 2008–2012 period. In addition to reducing emissions from fossil fuel burning, the Kyoto Protocol provides explicit opportunities for Annex B countries to partly achieve their reduction commitments by planting new forests, or by managing existing forests or agricultural land differently (so-called Land-Use, Land-Use Change and Forestry measures: LULUCF). The presumption of these LULUCF options is that removing CO_2 _from the atmosphere can also contribute to the stabilization of the atmospheric CO_2 _concentration and thus to a limitation of climate change. After the Kyoto Protocol was signed, a number of technical issues regarding the use of carbon plantations in achieving the country commitments remained open. For example, it has been unclear how to quantify the LULUCF potential, both in the short and the long terms. Furthermore, criticism on establishing new forests (so-called carbon plantations) as a mitigation strategy were related to the permanency of sequestration and whether the sequestration is additional to default developments (e.g. [6]). Permanency is uncertain, since the pressure on land for other purposes than carbon plantations may increase considerably in the near future along with shifts in disturbance regimes. The Food and Agriculture Organization of the United Nations (FAO), for example, projects considerable increases in arable land needed for food production [7], whereas land requirements for modern biofuels are increasing considerably as well [8]. Furthermore, the Kyoto Protocol clearly states that activities should not be in conflict with existing conventions, such as the Convention on Biological Diversity. Thus land-use changes that drive losses in biodiversity should be prevented [9].

The Kyoto Protocol has resulted in several studies estimating the sequestration potential in plantations. The IPCC's special report on Land use, land-use change and forestry (LULUCF), for example, suggests that there is a potential to sequester an additional 87 Pg C by 2050 in global forests alone [10]. Other studies even suggest that land-based mitigation could be cost-effective compared to energy-related mitigation options, and could provide a large proportion of the total mitigation [11,12]. However, it is often difficult to compare the results of these studies because they differ in terms and definitions and methods used. Furthermore, studies determine the sequestration potential in specific regions or specific land-cover types (e.g. [13-15]). Finally, there are studies that incorporate crude assumptions for future land-use change. For example, Sathaye et al. [16] based their projections of C sinks on linear extrapolation of continuing deforestation and afforestation rates, whereas Sohngen & Sedjo [17] only considered an increase in forest product demand, discarding future food demand.

The main objective of this paper is to present a methodology that quantifies the possible role of C plantations around the world in mitigating the build-up of CO_2 _in the atmosphere at different cost levels and assumptions; it also takes into account the aforementioned limitations and concerns. We specifically address the issue of net carbon sequestration, including the continued carbon sequestration of the original natural vegetation. Moreover, we only consider the carbon sequestration potential in regions that are not used for other ecosystem services (like food supply), and include future land-use change. In this study we use the methodology as being implemented in the IMAGE-2 model (Integrated Model to Assess the Global Environment [18]) to show the long-term potential in eighteen different world regions.

## Results

We present the global and regional distribution and C uptake potential of plantations for the different experiments and scenarios up to 2100 (see methodology section for detailed definitions of the different potentials). First, the physical potential is given (Experiments 1, 2 and 3), which is the potential based on local physical, ecological and environmental conditions. Second, the physical potential is translated into a social potential by taking interference with food and wood availability and nature conservation as main limitations (Experiments 4, 5 and 6). This is a general attempt to simulate societal barriers to the establishment of plantations that can also include other, such as, for example, institutional factors. These factors differ between regions, and hence the uncertainty within our projected "social potential" may be larger than that within the physical potential. The final step 3 (= economic potential, including also land and establishment costs) is described in detail in Strengers et al [19], including the sequestration potential. The experiments differ with respect to the used management of the carbon plantations and baseline scenarios used. The latter refer to the IPCC SRES A1b and B2 baseline scenarios [20] (see section on Model application for differences between these scenarios). Regarding management, the carbon plantations are either harvested at regular intervals or not harvested at all (called permanent carbon plantation). These management options can have a considerable effect on the uptake potential of plantations (see methodology section).

### Experiments 1, 2 and 3: Physical potential of carbon plantations

**Figure 1:**
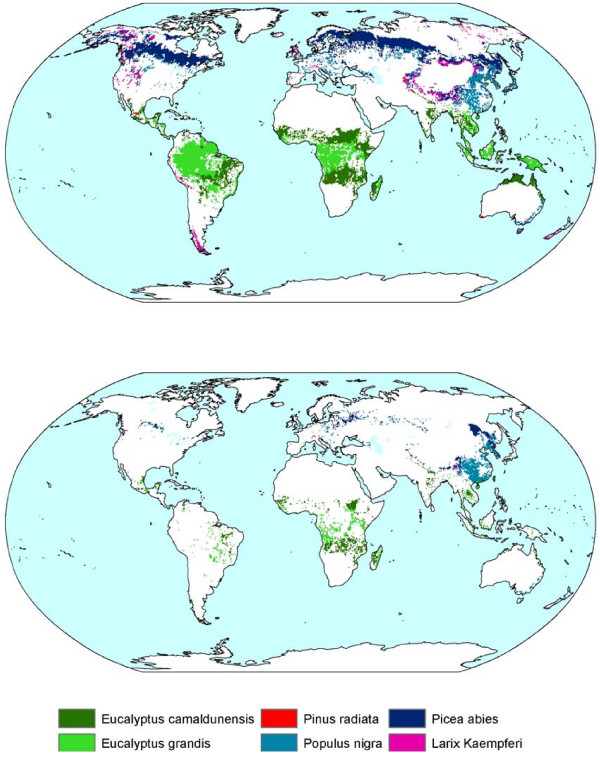
Physical (top) and social (bottom) potential distribution of permanent carbon plantations in 2100 using the A1b scenario.

In these experiments carbon plantations are established wherever they can grow and wherever they are carbon-effective compared to the baseline. Under this assumption, the six plantation types are found to be effective over large areas around the world (Figure 1). Under the A1b baseline scenario, about 3990 and 3850 Mha (i.e. 10^10 ^m^2^) plantations can be established under the permanent and frequent-harvest management options, respectively up to 2100 (Table 1). Plantations of gum species (*Eucalyptus *spp.), for example, are projected for establishment mainly in regions that are currently covered by savanna, woodland and even some tropical forest. The potential over the next few decades is limited because much land is needed for agricultural production (this land cannot be used because of the assumption that current and future agricultural land is to be excluded). Under the alternative B2 baseline scenario less land is projected to become available for plantations than under the A1b baseline, due to greater demand for agricultural land. The projected difference between the two management options (i.e. harvested or permanent plantations) has two reasons. First, the difference results from the assumption for permanent plantations that abandoned agricultural land is not available if the re-grown natural forest is used at a later stage to fulfill the wood demand. Second, close to 2100 permanent plantations are estimated to be more widely distributed because the CO_2 _emissions related to the harvest of plantations need to be compensated before harvested plantations become an effective C sink.

**Table 1:** Physical potential distribution of carbon plantations (in Mha).

**Baseline**	**A1b Permanent**			**A1b Harvest**			**B2 Harvest**		
	2030	2050	2100	2030	2050	2100	2030	2050	2100

River red gum	545	620	965	621	700	997	514	533	701
Rose gum	790	814	1310	1027	1039	1257	906	939	1157
Radiata pine	20	25	33	20	25	33	22	30	38
Black poplar	86	121	445	151	236	434	146	206	436
Norway spruce	792	845	984	778	828	855	1047	1141	1254
Japanese larch	100	158	254	128	183	272	139	195	247

**Global total**	**2333**	**2583**	**3992**	**2726**	**3011**	**3848**	**2774**	**3044**	**3833**

**Figure 2:**
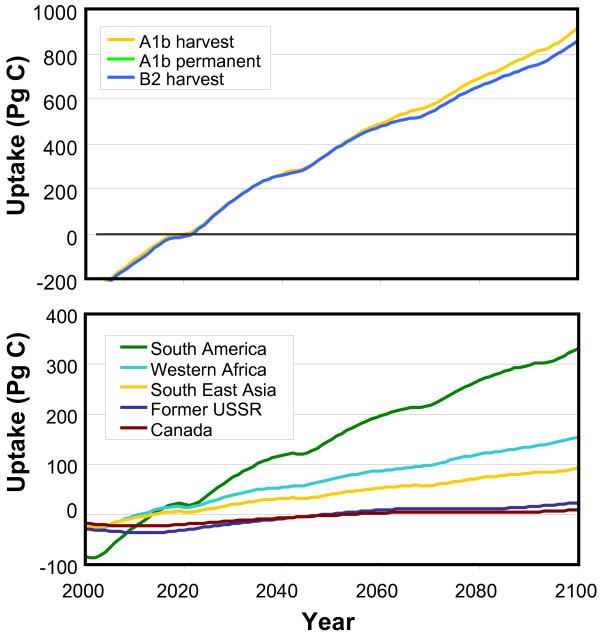
**Cumulative physical global (top) and regional (bottom) C sequestration potential (CSeq)**. The regional figure illustrates the trend in the A1b harvest experiment (in Pg C).

The projected cumulative physical C sequestration of plantations in the A1b scenario is 583 Pg C and 913 Pg C up to 2100 for the permanent and harvest options, respectively (Figure 2). Under the B2 baseline scenario, the cumulative potential is estimated to be 858 Pg C, considering frequent harvests (i.e. 6% less compared to A1b). These uptake rates equal about 37% and 58% of the projected overall CO_2 _energy and industry emissions in the A1b scenario for the permanent and harvest options, respectively. Under the B2 baseline, the estimated uptake is even 67% of the energy and industry emissions. Hence, the projected long-term physical potential of carbon plantations for slowing down the atmospheric CO_2 _increase is large. However, it will take more than 20 years to compensate for carbon emissions related to the establishment of the plantations. The projected physical potential up to 2020 is negligible where the cumulative potential up to 2030 is about 100–150 Pg C (Figure 2).

The two management options show a higher C sequestration potential in the case of harvested carbon plantations, especially beyond 2050 (Figure 2). This is caused by a decreasing sequestration rate for permanent carbon plantations, whereas the uptake potential remains high if a carbon plantation is frequently harvested harvests. This difference is induced by the C sequestration of plantations decreasing with age. The average age increases in permanent plantations but remains low in the frequent harvest case. This difference is projected specifically for plantations in Latin America and Africa.

Geographically speaking, the highest physical sequestration rates have been projected for plantations in tropical regions like South America and Africa, dominated by the two Eucalyptus plantation types (Figure 2). The projected sequestration potential is relatively low in high latitudes, because of low growth rates. In various parts of Canada and Russia, the net cumulative carbon sequestration even remains negative for about 50 years.

### Experiments 4, 5 and 6: Social potential of carbon plantations

We assessed the social sequestration potential of C plantations up to 2100 using wood availability and nature conservation as main constraints in addition to the food security criterion. These constraints have been implemented by estimating the potential on abandoned agricultural land only. Assuming permanent carbon plantations (Experiment 4), 181 and 831 Ma are projected in the A1b scenario potentially to be established around the world up to 2050 and 2100, respectively (Table 2). In the case of harvested carbon plantations, the area available in 2100 is projected to be 1014 and 695 Mha under the A1b and B2 baseline scenarios, respectively (Experiments 5 & 6). The difference between the baseline scenarios is caused by a larger land abandonment under the A1b baseline scenario than under the B2 baseline. The difference between the two management options is caused by the assumption for permanent carbon plantations that abandoned agricultural land is not available if the re-grown natural forest is needed at a later stage to fulfill the wood demand. For frequently harvested carbon plantations, the timber from the plantations is used to fulfill the wood demand, reducing the pressure on existing forests. Similar to the physical potential, the difference between the management options is projected to decrease near to 2100 because the CO_2 _emissions related to the harvest need to be compensated before the plantations become an effective C sink. As a consequence, fewer harvested plantations will be established.

**Table 2:** Social potential distribution of carbon plantations with establishment on abandoned agricultural land only (in Mha).

**Baseline**	**A1b Permanent**			**A1b Harvest**			**B2 Harvest**		
	2030	2050	2100	2030	2050	2100	2030	2050	2100

River red gum	31	75	317	33	83	332	30	37	158
Rose gum	20	26	230	30	41	256	21	34	108
Radiata pine	2	2	3	2	2	3	2	2	3
Black poplar	16	24	192	23	83	219	31	84	163
Norway spruce	31	48	75	128	164	181	119	203	234
Japanese larch	4	5	14	11	16	22	15	23	29

**Global total**	**105**	**181**	**831**	**228**	**390**	**1014**	**218**	**383**	**695**

*Canada*	2.2	6.7	14.8	16.2	27.2	26.4	15.9	36.2	30.2
*US*	0.9	0.9	2.9	5.0	5.2	5.1	18.4	53.2	69.0
*Europe*	0.3	0.9	19.5	4.3	12.2	24.7	17.4	36.5	35.4
*FSU*	13.7	20.5	49.9	48.7	67.1	79.9	80.6	107.1	135.8
*China*	0.3	14.9	187.5	3.0	60.8	254.7	0.0	25.2	125.2
*Latin America*	40.0	47.7	102.9	76.3	85.9	133.3	35.5	56.6	63.8
*Africa*	6.4	43.5	314.6	10.5	61.1	326.1	0.4	1.6	124.8
*India*	0.4	0.4	54.8	0.6	0.6	56.5	1.2	1.2	27.2
*SE-Asia*	0.0	0.0	27.6	0.0	0.9	30.4	0.0	0.0	7.2
*Oceania*	41.0	45.7	53.8	63.5	69.0	74.5	47.7	65.5	71.5

**Figure 3:**
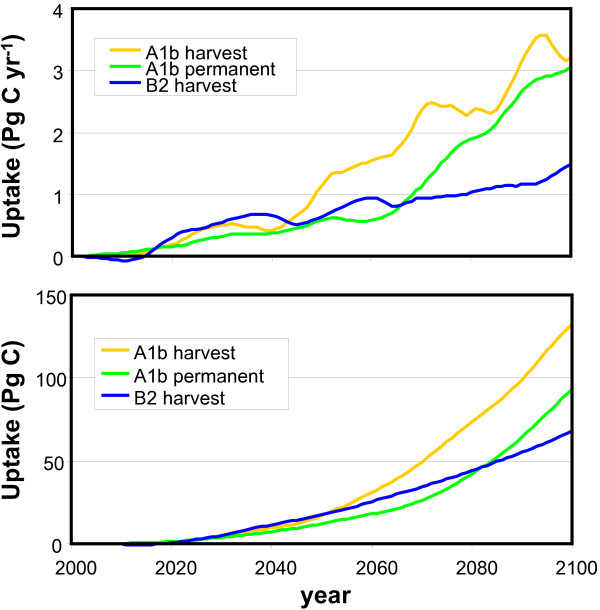
**Social C sequestration potential (CSeq) on abandoned agricultural land**; top – annual (Pg C/yr), bottom – cumulative (Pg C).

The majority of the carbon plantations is projected in all the experiments to be established after 2050, because land only becomes available then, due to decreasing population and increasing efficiency. The projected cumulative global social C sequestration potential remains low in the coming decades (Figure 3), and, up to 2050, reaches 12–17 Pg C for the different baselines and harvest regimes (Table 3). Under the A1b scenario the potential increases up to 93 and 133 Pg C in 2100 for permanent and harvested plantations, respectively (Figure 3 and Table 3). This is 5–7% of the projected cumulative emissions up to 2100 coming from the energy and industry sector (i.e. about 1740 Pg C). The potential uptake up to 2100 under the B2 scenario is 68 Pg C, implying 5% of the energy and industry emissions (i.e. 1272 Pg C). The net C sequestration potential can be higher under a frequent harvest regime due to a higher area-based uptake and the broader distribution. Comparing the 2 baseline scenarios, the projected global sequestration of carbon plantations in 2100 is 95% higher under the A1b scenario than in the B2 baseline (Table 3). This is mainly due to the higher establishment rates.

**Table 3:** Implications of establishing carbon plantations on abandoned agricultural land.

Indicator	2050			2100		
	A1b perm.	A1b harvest	B2 harvest	A1b perm.	A1b harvest	B2 harvest
Baseline atmos. CO_2 _concentration (ppm)	561	561	506	753	753	606
Change in CO_2 _concentration, compared to baseline (ppm)	-5	-6	-8	-39	-52	-27

Cumulative social C sequestration potential in C plantations on abandoned agricultural land only (Pg C)						
**Global potential**	**12**	**17**	**17**	**93**	**133**	**68**
*Canada*	0.2	0.6	0.8	0.7	1.7	1.9
*US*	0.1	0.3	1.7	0.3	0.6	7.5
*Europe*	0.1	0.4	1.5	0.7	2.3	3.6
*FSU*	0.6	1.9	3.9	3.4	5.2	7.7
*China*	0.1	0.8	0.2	10.3	26.3	10.3
*Latin America*	4.2	5.0	3.8	13.6	18.2	13.0
*Africa*	2.8	3.5	0.1	47.9	57.6	8.1
*India*	0.0	0.0	0.2	6.5	6.8	2.9
*SE-Asia*	0.0	0.0	0.0	0.2	2.8	0.7
*Oceania*	4.0	4.4	5.1	9.8	11.2	12.3

Geographically speaking, most plantations are projected for establishment in tropical regions (Figure 1 and Table 2). The consequences for the C sequestration are that under the A1b baseline scenario, 40–50% of the global potential can be sequestered in plantations in Africa, 10–20%, in China, 10% in Latin America, and 10% in Oceania (Table 3). Although a considerable amount of abandoned agricultural land is projected for Europe, Canada and the FSU as well, the effectiveness of establishing C plantations here is projected as being rather limited. For example, 6% of the global potential area can be established in the FSU up to 2100, sequestering only 4% of the global potential.

With respect to the social potential, evaluating the effectiveness of carbon plantations in slowing down the build-up of CO_2 _in the atmosphere shows that the concentration in 2100 under the A1b scenario can be reduced from 752 to 713 ppm (i.e. a 39 ppm reduction) when planting permanent carbon plantations, whereas it reaches 700 ppm (i.e. a 52 ppm reduction) assuming frequently harvested plantations (Table 3). The two management options differ because of the broader distribution of carbon plantations when planting frequently harvested plantations and because of the additional C that will be stored in the soil compartment. The lower social sequestration potential projected under the B2 baseline scenario results, obviously, in a lower effectiveness. Assuming frequently harvested carbon plantations, we project a CO_2 _concentration of 579 ppm in 2100, which is 27 ppm less than in the baseline.

## Discussion

### The carbon sequestration potential in comparison with other studies

Here we have presented a methodology to assess the global and regional sequestering potential of carbon plantations established after 2000. Based on ecological and environmental constraints alone, carbon plantations can be effective in large parts of the world with a projected cumulative sequestering potential of 913 Pg C up to 2100. In the A1b baseline scenario this equals 52% of the total cumulative CO_2 _emissions from energy and industry from 2000 to 2100. In the B2 scenarios it is even 67%. The social sequestration potential is much lower but still considerable. The annual average global potential is projected at 0.1 – 0.2 Pg C yr^-1 ^up to 2050, and 0.68–1.3 Pg C yr^-1 ^up to 2100 (Table 3). In 2100 this leads to a 27–52 ppm smaller increase in the atmospheric CO_2 _concentration and compensates for 5–7% of the total energy and industry related CO_2 _emissions. The sequestration potential is likely to considerably increase beyond 2100, because many plantations are projected to be established only close to the end of the 21^st ^century. This holds especially for regions where large areas of arable land are expected to be abandoned towards 2100, such as China.

The social sequestration potential of the plantations projected up to 2050 is at the low end of ranges found in the literature, whereas values for the coming 100 years are more in line (Table 4). Geographically, the most effective plantations are located in tropical regions, whereas due to low growth rates the C sequestration in high latitudinal plantations is limited (Table 3). This is in line with the findings of Masera et al. [21] and Cannell [22]. Many other estimates are especially useful in a comparison with our area-based potentials, because the studies often focus on the C sequestration potential in existing forests (Table 4). For example, the projected social C sequestration potential of tropical plantations of Latin America and Africa (1.6–1.9 Mg C ha^-1 ^yr^-1 ^for 2000–2100), is found at the low end of the range given by Silver et al. [23]. Our projections for Europe up to 2100 – between 0.3 and 1.1 Mg C ha-1 yr-1 – are well in line with the projected area-based uptake of 0.52 Mg C ha-1 yr-1 given by Liski et al [24]. Note that we have used a particular definition of "social potential", possibly causing differences with other studies in either direction. Areas that we excluded, for example, because of competition with other needs may be converted in reality, while areas that we included could not be appropriate for the establishment of plantations because of other social or institutional factors.

**Table 4:** Comparison of existing C sequestration projections.

**Reference**	**Total C sequestration (Pg C yr^-1^)**	**Areal C sequestration (Mg C ha^-1^yr^-1^)**	**Period**	**Remarks**
**Global studies**				

This study (social potential)	0.12 – 0.17 0.68 – 1.33	0.9 – 1.3 0.8 – 1.3	2000–2050 2000–2100	Considering sequestration on abandoned agricultural land only
[10]	0.2–0.58		2008–2012	
[22]	0.2 – 1		2000–2050	Conservative potential for 50-year period
[55]	0.3–2.9	0.8–1.6	2000–2075	Large variation due to different assumptions on yields
[61]	avg 1.04		1995–2095	
[62]	0.15–0.8		2008–2012	
[63]	0.6–1.2		2000–2050	Only in degraded land soils. Total potential is 30–60 Pg C.

**Regional studies (Compared to Table 3)**				

[22]	0.02–0.05			Europe, a 100-year period
[23]		2–3.5		Average sequestration of tropical forests during an 80-year period
[55]				Only above-ground sequestration. soil decomposition fluxes excluded
		0.6–1		Canada
		0.5–11		USA (many studies summarized)
		1.4–2.3		Western Europe
		7.5–7.7		Australia
[64]	0.006		2010	EU25 countries
	0.01		2020	
	0.02		2030	
[65]	0.05 0.12		2100	EU15. only soils Wider Europe (excl. Russia). only soils
[66]		0.3–0.6		European forests during 2008–2012
[67]		0.35		North-west Russia
[68]		1.4	1999–2000	Canada
[69]	0.88	0.3	Current	Sink of all boreal and temperate forests
	0.11	0.52		All European forests
	0.43	0.48		All Russian forests
	0.10	0.25		All Canadian forests
	0.17	0.56		All US forests

Despite the estimated considerable C sequestration potential up to 2100, the uptake potential for the coming decades is projected to be limited (Figure 3). It can take about 20 years to compensate for the emissions related to the establishment of the plantations. Moreover, not much agricultural land will likely be abandoned in coming decades due to the current and projected agricultural pressure. The limited potential in coming decades is in line with findings of Marland & Schlamadinger [25], who showed that the sequestration potential in forests established since 1990 is mainly relevant in the long term. As such, we do not confirm the suggestion of Kirschbaum [26] that plantations may help to buy some time in initiating emission reductions already in the next few decades.

The limited role of plantations in the coming decades might be caused by our assumptions that C plantations can only be established after 2000. Various other studies report afforestation activities in different locations around the world, even before 2000. Brown [27] and FAO [28], for example, reported that globally 124 Mha and 187 Mha forest plantations have been established up to 1995 and 2000, respectively. More than 90% of these plantations have been established in 30 countries only, mainly in such Asian countries as China (45 Mha), India (32 Mha), and Japan (11 Mha). Furthermore, various studies report existing afforestation activities, but seldom account for deforestation in the same region (the so-called leakage effect). This has also been shown by others (e.g. [29]) by estimating an annual afforestation rate in the tropics of 2.6 Mha yr^-1 ^throughout the 1980s, but at the same time a deforestation rate of 15.4 Ma yr^-1^. In our methodology, leakage is not possible because we only establish plantations on land that is available for the entire simulation period (i.e. up to 2100). Finally, our projections are lower than in other studies that account for the C sequestration in forests planted for various other reasons (e.g. recreation, agroforestry and soil restoration). For India, for example, we have project a negligible afforestation potential up to 2030 because of the large pressure on the land for food production. Nevertheless, Ravindranath & Somashekhar [30] reported an afforestation rate of India of 1.6 Mha yr^-1^, mainly for agroforestry purposes. Again, these afforestation rates are partly counterbalanced by deforestation activities in India [30,31].

### The methodology in relation to conventions and protocols

The methodology presented is aimed at quantifying the sequestration potential of carbon plantations around the world, in consideration of the requirements mentioned in different conventions and protocols. The UN Framework Convention on Climate Change [3] and its underlying Kyoto Protocol, which opened the possibility for developed countries to use afforestation programs in achieving their reduction commitments, clearly stress that C plantations are only effective in the long term if (see also [10,32,33]):

• they are *additional *to a baseline;

• all C fluxes are considered (i.e. *full C accounting);*

• they are *permanent*. If not, a carbon plantation has little value in terms of actually reducing the concentration of GHG in the atmosphere, since carbon sequestered over various years will return to the atmosphere;

• the credited C sequestration in one region is not to be compensated by C losses elsewhere (i.e. no *leakage *[34]),

• the C sequestration in plantations exclude 'indirect human influences' in terms of, for example, climate and CO_2 _change.

The *additionality *issue has been taken into account in the methodology presented by considering the sequestration potential of both plantations and natural ecosystems. Furthermore, the methodology considers *all C fluxes *by keeping track of fluxes in both vegetation and soil, plus the carbon losses due to the establishment of the plantations. The *permanency *concern is taken into account by comparing the C plantation option with various other land-use options. Alternative land-use options pose a main threat to the permanency of a carbon plantation, especially in the long term (e.g. when the demand for agricultural land fluctuates or prices of land-use products change). Since permanency is more certain if plantations are established in areas that are not used for food, fodder and timber production, areas needed for agriculture or wood up to 2100 have been excluded in the all experiments. As mentioned earlier, leakage is not possible in the methodology presented because we only establish plantations on land available for the entire simulation period (i.e. up to 2100). Finally, the methodology accounts only for carbon sequestered *directly *by the plantations, corrected for climate change and CO_2 _fertilization (i.e. indirect human influences). This has been done both for the historical uptake – where we corrected 1995 growth rates for observed changes in CO_2 _and climate (see Equation 2) – as well as the projected future (reducing the projected social potential in the supply curves for climate and CO_2 _changes in the baseline).

### The effectiveness of carbon plantations in a broader environmental context

The effectiveness of harvesting plantations and using the biomass to displace fossil fuels and/or timber, compared to having carbon stored in a permanent plantation, depends to a great extent on the displacement factor (i.e. the extent to which wood from carbon plantations can be effectively used to replace fossil fuels) [35]. Here, a displacement factor of 'one' is assumed. Theoretically this can be achieved if fossil fuels are displaced by harvested wood [22,36]. However, if the displacement factor is (much) smaller than 'one', the environmental effectiveness of harvested plantations decreases sharply. Likewise, establishing carbon plantations is, in general, less effective than avoiding deforestation (especially in tropical regions, [37,16]). This, however, is associated with various social difficulties and avoiding deforestation in one region may be counterbalanced by additional deforestation elsewhere.

The effectiveness of carbon plantations in especially high latitudes is questioned because of the effect on different biophysical processes (i.e. changed radiation balance) that may counterbalance the additional C sequestration [38-40]. On the basis of the albedo effect and the projected low net sequestration potential for high latitudinal plantations (i.e. in parts of Canada and Russia the net C sequestration even remains negative for about 50 years), the establishment of carbon plantations in high latitudes is only favorable if the objective to sequester carbon is combined with other environmental considerations. For example, plantations may also contribute to water protection and soil erosion control [21,41].

An environmental constraint often mentioned for large-scale C plantations is the availability of water and nitrogen [41-43]. Also in the methodology presented, the high growth rates of the carbon plantations (compared to natural forests) rely on a high level of management, including nitrogen fertilization for plantations situated on poor or degraded soils. The additional use of water and fertilizer should indeed be a concern in the planning and management of the plantation, especially because a (higher) fertilizer use could imply additional emissions of N_2_O, which were neither accounted for in our study, nor in most other studies. Likewise, afforestation activities have recently also been questioned in the context of possible additional methane emissions from trees – the second-most important greenhouse gas [44]. Although this issue is currently still under scientific debate, the effectiveness of afforestation programs would be reduced by a maximum of 10%. This has been confirmed by others (see, for example, [45] for a more detailed discussion).

## Conclusion

We have presented a rule-based methodology to quantify the long-term physical and social sequestration potential of carbon plantations up to the end of the 21^st ^century and their effectiveness in slowing down the increase in atmospheric CO_2_. Applying the methodology, we conclude that projected potentials differ considerably for different experiments, regions and management options. For example, we projected a nearly 100% difference in the sequestration potential up to 2100 between two baseline scenarios, showing the effect of uncertainties in future land use. Nevertheless, in all cases the C sequestration potential can be substantial. Even under a conservative set of assumptions, the cumulative sequestration potential up to 2100 compensates for 5–7% of the total energy and industry related CO_2 _emissions. But the sequestration potential is substantial only in the long term. The potential for the coming decades is limited due to the limited amount of available land and the long period needed to compensate for emissions related to the establishment of the plantations. Geographically speaking, plantations in tropical regions are most effective. The C sequestration potential of plantations in high latitudes is low and because of biophysical feedbacks on the climate system its effectiveness can even be questioned. The establishment of plantations in these regions is only favorable if the objective to sequester carbon is combined with other environmental considerations.

Finally, our analysis showed that C sequestration in plantations may be substantial and thus can help to slow down the future increase in atmospheric CO_2_. But C plantations do not represent the ultimate solution to the problem of establishing a stabilization of the atmospheric CO_2 _concentration. They should form part of a broader package of options, with clear measures for also reducing energy emissions.

## Methodology

### The algorithm

**Figure 4:**
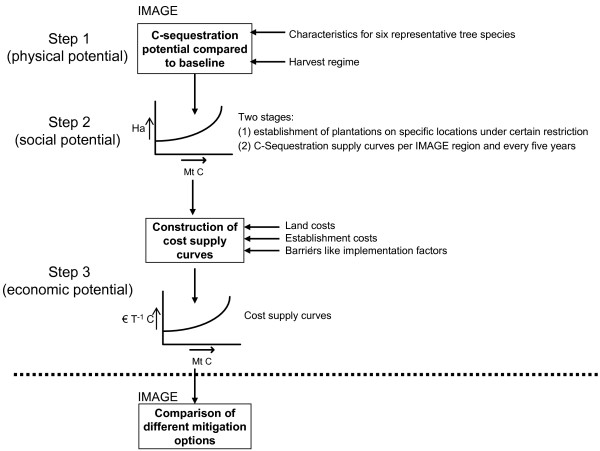
Steps to quantify sequestration potential of carbon plantations.

The methodology to assess the C sequestration potential in carbon plantations, as presented here, is a rule-based approach that is implemented on a geographical explicit -0.5° longitude × 0.5° latitude-grid (Figure 4). The time horizon is 2000 – 2100. This facilitates the quantification of the long-term potential of carbon plantations in different parts of the world in mitigating the build-up of CO_2 _in the atmosphere. We distinguish different potentials, defined according IPCC definitions [46]. The methodology consists of three steps (Figure 4). The first step is to determine the *physical sequestration *potential of C plantations, accomplish by adding carbon plantations as a new land cover class in IMAGE 2 (see below for a general description of the IMAGE 2 model). All carbon pools and fluxes of the potential carbon plantations (e.g. Net Primary Production – NPP and Net Ecosystem Productivity – NEP) are calculated by the IMAGE-2 terrestrial C-cycle model, taking environmental (e.g. climate and atmospheric CO_2_) and local conditions (e.g. soil) into consideration. In the second step, the *social potential *of plantations is determined using the restriction 'no interference with food supply and nature conservation'. In the third step, the social potential is transferred into the economic potential by linking the C sequestration potential to establishment and land costs. The resulting marginal abatement cost curves can be used to compare the potential of carbon plantations with other mitigation strategies using cost minimization (e.g. [47]). The focus of this paper is on describing and analyzing steps 1 and 2 of the methodology. We will also summarize step 3 (i.e. economic potential), but refer for details on this to the companion paper by Strengers et al. [19].

#### Step 1: The physical sequestration potential

The starting point for this step is the *potential distribution *of C plantations around the world. Six plantations types were selected on the basis of the 'Top 14 Most Planted World's Trees' [28,48] to represent suitable species in different climatic zones around the world (Table 5). We used, for example, gum species (*Eucalyptus *spp.) for the tropical regions, and spruce (*Picea abies*) and larch (*Larix kaempferi*) for plantations in cool and boreal regions, respectively. 'Potential distribution' in this context refers to the *availability *and *suitability *of land. Land is assumed to be *available *when it is not assigned as protected area and no longer used for agriculture (neither cropland nor pasture). Hence, a more realistic potential is provided, given the many other land-use purposes that may expand in the (short-term) future. *Suitability *of land is driven by various environmental conditions in terms of climate and soil. All these conditions need to be fulfilled to allow a specific plantation type in a certain region. The climatic characteristics of the plantations are derived from the best matching Plant Functional Types (PFT) – classes of plant species grouped according to physiological characteristics and the sensitivity to changes in temperature and water availability (Table 5).

**Table 5:** The climatic characteristics of the selected tree species for carbon plantations.

**No**	**Tree species**		**Corresponding PFT**	**T_cold _(°C)**	**Moisture^2^**	**GDD5_min_**
1	Eucalyptus camadulensis	River red gum	Tropical deciduous trees	>15.5	0.45 to 0.8	
2	Eucalyptus grandis	Rose gum	Tropical evergreen trees	>15.5	0.8 to 1.0	
3	Pinus radiate	Radiata pine	Temperate evergreen trees	>5	0.55 to 0.95	
4	Populus nigra	Black poplar	Temperate deciduous trees	-15 to 15.5	0.65 to 1.0	1200
5	Picea abies	Norway spruce	Boreal evergreen trees	-35 to -2	0.75 to 1.0	350
6	Larix kaempferi	Japanese Larch	Boreal deciduous trees	< 5	0.65 to 1.0	350

^1 ^T_cold _is the average temperature of the coldest month.

^2^Moisture is expressed as the ratio between actual and potential evapotranspiration [70]. The lower end of the range may decrease due to increasing Water Use Efficiency. This is the result of increasing atmospheric CO_2 _levels.

^3 ^GDD5_min _is the minimum degree-day sum for establishment (considering a 5°C base).

Secondly, the best growing plantations out of these six types are determined for each grid cell by using the parameters describing the C dynamics (e.g. lifetimes, allocation fractions). These parameters of the different plantation types are linked to the parameters used for the natural land-cover type that best matches the plantation type considered (Tables 5 &6; [49,19]). The Net Primary Production (NPPCP_ts_) rates averaged over the longest likely rotation length (LRL) of each plantation type (Equation 1) are compared. The longest LRL has been chosen to take into account the period needed to reach the maximum NPP for all possible plantation types.

**Table 6:** The carbon characteristics of the selected tree species for carbon plantations.

No.	Corresponding land cover types	Yield (m^3^/ha yr)	Recov. (yr)	LRL^1 ^(yr)	HI^2 ^(-)	WD^3 ^(Mg DM/m^3^)	FNPP_CP _(Mg C/ha yr)	AGF (-)	CF95_ts _(Eq. 2)
1	Trop. deciduous forest	12 (3–20)	8	15	0.65	0.550	18.9	2.02	1.041
2	Trop. evergreen forest	20 (10–35)	8	15	0.70	0.425	22.2	1.77	1.042
3	Warm mixed forest	14 (10–30)	15	28	0.87	0.450	11.0	1.62	1.045
4	Temp. deciduous forest	16 (8–28)	18	25	0.83	0.350	11.8	1.77	1.022
5	Cool mixed forest	11 (4–20)	30	60	0.87	0.400	8.2	1.49	1.00
6	Boreal forest	7 (4–12)	25	60	0.87	0.490	5.6	1.11	1.00

^1 ^Likely Rotation Length: derived from [28] for both eucalyptus plantations; pine average of [48; 61; 71]; poplar based on [48; 71]; spruce based on [61] and larch derived from yield tables (e.g. [72; 73]), use the moment that growth rates start to decline.

^2 ^Harvest Index-based [34]

^3 ^Wood density mainly based on [34]; If not available, use [71; 74; 75; 76].

*NPPCP*_*ts*_(*t*) = *RF*(*t*)·*FNPP*_*lct*(*ts*)_(*t*)·*AGF*_*ts*_

where

ts Index for tree species in a carbon plantation (1,..,6)

lct(ts) Land cover type by which the carbon dynamics of tree species (ts) are described (Table 1)

RF(t) Reduction Factor (≤1) during the period towards maximum average growth in terms of NPP, i.e. the recovery time (-) (Table 1)

FNPP_lct(ts)_(t) NPP of full-grown natural vegetation in year t if the grid cell were to be covered by land-cover type lct(ts), as computed by the IMAGE 2 C cycle model (Mg C ha-1 yr-1)

AGF_ts _Additional Growth Factor of tree species ts (-), (Table 1, Equation 2)

The additional growth factor (*AGF*_*ts*_, Equation 2) is defined as the growth rate of a plantation – based on a literature review (Equation 3) – compared to the average growth of the natural land-cover type, corrected for historical environmental changes – *CF95*_*ts*_. The latter correction factor is needed because the information taken from the literature on the NPP of plantations comprised, in general, data from around 1995. Following the rules in the Kyoto Protocol – stating that sequestration credits should only be based on 'direct human activities' – the NPP data needed to be adjusted. This is because these data include a growth stimulus caused by, among other factors, increasing CO_2 _concentrations (which form 'indirect human activities'). The *CF95 *value for each plantation type (Table 5) has been derived by applying the IMAGE-2 C-cycle model in order to define the growth stimulants from CO_2 _and climate since 1970. Note that we correct the sequestration potential up to 2100 in a similar way:

$$AGF_{ts}=\frac{FNPP_{CP,ts}}{NPPI_{lct(ts)}\cdot CF95_{ts}}$$

where

FNPP_CP,ts _Average NPP of full grown plantations (Mg C ha-1 yr-1) around 1995 (Eq. 3)

NPPI_lct(ts) _Average NPP of all grid cells in 1970 covered by land-cover type lct(ts) (Mg C ha-1 yr-1) [19]

CF95_ts _Correction Factor for climate-induced growth stimulants for 1970–1995 (-).

*FNPP*_*CP *_can be derived from especially literature on plantations yields ([27,28,34,50,51] and Table 6). This information is subsequently used in Equation 3 (see also [19])

F=(ASLS−ABLB)+(0.5−2ASLS−2ABLB)FNPPCP=YLD⋅WD⋅CF⋅LRLHIF⋅(LRL−Re⁡cov⁡2)

where

AS Allocation Fraction of Stems (= 0.3)

LS Lifetime of stems, based on the underlying land-cover types lct(ts) (yr)

AB Allocation Fraction of Branches (= 0.2)

LB Lifetime of branches, based on the underlying land-cover types lct(ts) (yr)

YLD Yield of a plantation averaged over a rotation (m^3 ^Fresh Volume ha^-1 ^yr^-1^); (Table 7)

**Table 7:** Comparison of plantation growth rates around the world (m^3 ^ha^-1 ^yr^-1^).

Species	This study	[27]	[28]	[34]	[48]	[61]	[71]	[77]	[78]
*E. camaldulensis*	18	6–38	15–30	15–30					4–34
*E. grandis*	28		15–50	15–50	30–35 (tropics) 16–30 (rest of world)	25			35–50
*P. radiate*	16	26	12–35	12–35	20–22		11–25	18–30	8–23
*Poplar spp.*	19	9–30			12–20		9–19		8–40
*Picea abies*	13	5–21				5–8	10–15		4–12
*Larix kaempferi*	8	5–14							4–12

WD Wood density (Mg dry matter.m^-3 ^fresh volume; see Table 1)

HI Average harvest index or the fraction of above-ground biomass used (Table 1) of which the remainder decomposes to humus (-)

CF Average carbon factor or carbon content (Mg C m^-3 ^dry matter)

Recov Recovery time or the average time for a carbon plantation to reach maturity in terms of NPP (yr) (Table 6).

The last part in determining the physical potential (step 1) is to estimate the net C sequestration (CSeq) potential of the best growing species in a grid cell. This calculation is based on the concept of SPP (Surplus Potential Productivity), as introduced by Onigkeit et al. [52]. The basic philosophy is to account only for the *net *C uptake of a plantation (Equation 4). This is calculated by using emissions associated with the conversion from natural land cover into a plantation and comparing the NEP flux of a plantation with the NEP flux of the natural vegetation that would otherwise grow in the area. As such, CSeq determines the *additionality *compared to the situation of having no plantations. Note that a negative value of CSeq corresponds to a biospheric uptake of carbon from the atmosphere. In our application, the NEP fluxes are simulated by the terrestrial C cycle model of IMAGE 2, taking into account NPP and soil respiration (see below).

$$CSeq=b\cdot E+\sum_{t=t_{0}}^{t=2100}\left[NEP(t)-NEP_{CP}(t)\right]$$

where

CSeq Net carbon sequestration in a grid cell in the period t0 through 2100 (Mg C ha^-1^)

t Year (between 2000 and 2100)

t0 Starting year of carbon plantations in a grid cell

NEP_CP_(t) Net Ecosystem Productivity of best growing tree species in a grid cell (Mg C ha^-1 ^yr^-1^)

NEP(t) NEP of the original vegetation according to the baseline scenario (Mg C ha^-1 ^yr^-1^)

E C content of natural vegetation before the conversion into a carbon plantation (Mg C ha^-1^)

b Burn factor of the initial harvest [either 0 or 1] (-)

The variables *E *and b account for carbon emissions related to the establishment of a carbon plantation. For plantations established on abandoned agricultural land, grassland or forest land just being logged, there is no clearing needed and 'b' is close to zero. When, however, an existing natural forest or woodland is converted into a carbon plantation, the original vegetation is assumed to be burnt entirely (i.e. *b *= 1), resulting in instantaneous emissions of carbon into the atmosphere. These emissions must first be compensated before a plantation is effective in mitigating the CO_2 _build-up in the atmosphere.

Since management can have a considerable effect on the carbon uptake potential of plantations [53,54], we included two possible harvest regimes. Either plantations are harvested at regular intervals or no harvest takes place at all. In the latter case, a plantation will grow to a stable level of carbon storage and a low additional C sequestration further in time in the soil. In the former case, a plantation is harvested at the moment of maximum C sequestration, (i.e. the NEP of a plantation averaged over the stand age starts to decrease), followed by re-growth. In our assessment the harvested wood from stems and branches is used to fulfill the wood demand. Leaves, roots and the non-harvested stems and branches enter the litter and humus carbon pools in the soil. The approach of displacing wood demand amounts to a displacement factor of 1 (assuming no leakage, i.e. no change in the wood sector).

**Figure 5:**
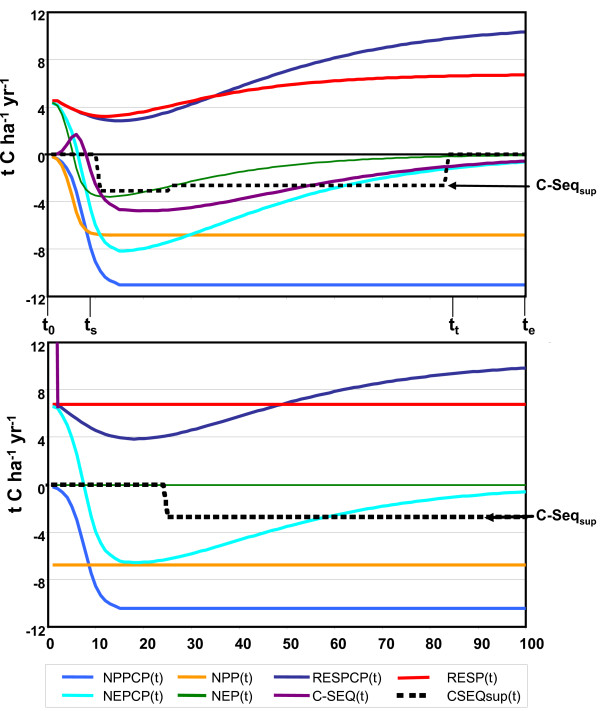
**Illustrative growth curves of a *Pinus radiata *plantation**: top – permanent plantation on abandoned agricultural land; bottom – permanent plantation on former forest area. Note that negative numbers represent a C uptake. Furthermore, the curves assume neither CO_2 _fertilization nor climate feedbacks.

Figure 5 illustrates step 1, showing the C dynamics of a *Pinus radiata *plantation on either abandoned agriculture or replacing a natural forest. In the case of establishing this plantation on abandoned agricultural land, the NPP of both the plantation and the natural forest – that would otherwise grow in the area – increases from zero up to the maximum value within the predefined recovery period. If responses to changing atmospheric CO_2 _levels and climate are excluded, the NPP values will remain constant at the maximum value. The soil respiration of both the plantation and natural forest first decline because the carbon input from young trees is limited, whereas the decomposition rate starts at the much higher equilibrium level with respect to the previous (in this case agricultural) vegetation. After a period of decline, the respiration flux increases, since the soil carbon pools are filled up again. The respiration flux increases until it exceeds NPP. If the net carbon uptake of the carbon plantation [*NEPCP(t)*] is larger than the net uptake of the natural forest [*NEP(t)*] (i.e. more negative), the plantation is effective in slowing down the build-up of atmospheric CO_2_. This is illustrated by negative values of *CSeq*. Since it is unknown in advance *when *a certain potential is actually used in a mitigation effort, we averaged the carbon sequestration over a predefined period of time expressed as *CSeq*_*sup*_. As such, the *CSeq*_*sup *_over the time interval [*t*_*s*_, *t*_*t*_] is an approximation of the average net carbon sequestration over the time interval [*t*_0_,*t*_*e*_].

In the case of the establishment of a C plantation on slash and burnt natural ecosystems (Figure 5), large quantities of carbon are emitted instantaneously (i.e. *E *will be large). Afterwards, *CSeq(t) *in year *t *equals *NEP*_*CP*_*(t)*, assuming no CO_2 _fertilization and other climate feedbacks (as such, the NEP of the natural vegetation is about 0). However, the year that a plantation starts to actually sequester carbon is postponed because the initial emissions have to be compensated (about 23 years for the example in Figure 5).

#### Step 2: The social sequestration potential

The social potential of the afforestation activities is estimated in two stages. Firstly, we establish plantations around the world using certain restrictions based on social acceptance. This is accomplished by using a particular definition of social importance: Considering only those areas that are neither needed for food and wood supply nor are covered by natural ecosystems (because of their importance for nature conservation). Establishing plantations on abandoned agricultural land is the only possibility. This leads to uptake potentials per grid cell (geographical explicit). Secondly, supply curves have been constructed for each IMAGE-2 region, summing-up the gridded sequestration potentials for all grid cells within that region where the average carbon sequestration, corrected for climate change and CO_2 _fertilization effects, is positive in a year 'z' (Figure 5, [19]). Since it is unknown when a certain potential is actually used in a mitigation effort, and to allow for comparison with other greenhouse gas mitigation options, the carbon sequestration is averaged over a predefined period of time (CSeq_sup_(t)). Thus each point in a supply curve represents the regional sum of the average annual carbon sequestration potential of a grid cell assigned to a time interval [*t*_*s*_, *t*_*t*_], starting with the most productive grid cells (i.e. cells with highest sequestration rate per hectare), ending with ineffective grid cells.

#### Step 3: The economic sequestration potential

The social C sequestration potential is used to determine the economic potential by linking it to costs (see [19] for details). This results in Marginal Abatement Curves (MACs) or cost-supply curves dependent on geographical-explicit environmental circumstances and possible future changes in land use. In general, the most important cost factor in producing or conserving carbon sinks is land [55]. In addition, we also consider establishment costs. Other types of costs are excluded because they are either low (e.g. maintenance costs), compensated by revenues from timber, or difficult to quantify [56]. Land costs are based on GTAP data [57] for land values of agricultural land around the world. Establishment costs, set at 435 US$ (1995) per ha, are uniform in time and space. This assumption is supported by the survey of Sathaye et al. [15]. The value of 435 US$ (1995) per ha is based on analyzing variations between the regions and the ranges within the regions.

### The IMAGE 2 model

The methodology presented has been implemented in IMAGE 2 (Integrated Model to Assess the Global Environment [18,58,59]). This is a multi-disciplinary, integrated assessment model, designed to explore causes and effects of global environmental change. IMAGE 2 integrates different land-use demands like food, fodder, biofuels and C sequestration. IMAGE 2 is global in application and integrates regional socio-economic (i.e. eighteen regions) and geographically explicit grid dimensions (i.e. 0.5^0 ^longitude by 0.5^0 ^latitude). Each grid cell is characterized by its climate, soil and land cover (natural ecosystems or agriculture). Because of the dynamic land use, the geographic explicit modeling and the global perspective, IMAGE 2 is very suitable for the presented methodology.

**Figure 6:**
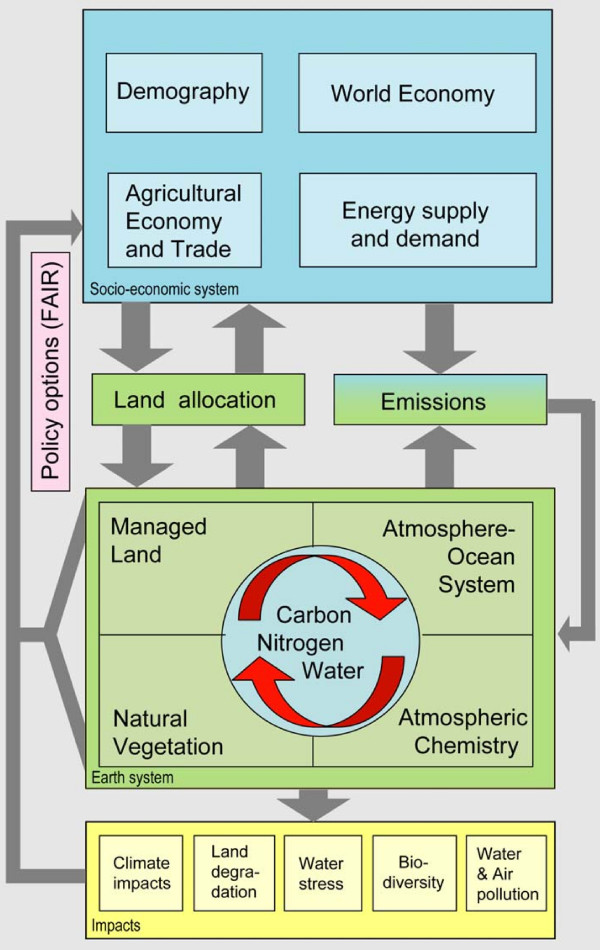
Structure of IMAGE 2.

IMAGE 2 consists of various sub-models (Figure 6). Drivers of the model are regional trends in wealth, demography and technology for the period 1970 to 2100. These trends determine, for example, the demand for land resources. Changes in production of or demand for land-related products (i.e. food, fodder, biofuel, timber and C sequestration) drive land-use changes, leading to land-use emissions of various greenhouse gases into the atmosphere. The IMAGE 2 atmospheric and ocean sub-model computes changes in atmospheric composition (e.g. CO_2 _and CH_4_) and, subsequently, the climate by using the land-use and energy-related emissions and by taking oceanic and terrestrial CO_2 _uptake and atmospheric chemistry into account. The climatic changes alter the distribution and productivity of ecosystems and agriculture, with both, in turn, affecting the terrestrial C dynamics.

Carbon plantations have been added as a separate land-cover class into the land-cover sub-model of IMAGE 2, whereas their carbon pools and fluxes are computed by the terrestrial C cycle sub-model [19,49,60]. The driving force of the C cycle sub-model is Net Primary Productivity (NPP), which is the photosynthetically fixed C in plants minus C losses due to plant respiration. NPP in IMAGE 2 is a function of atmospheric CO_2 _concentration, climate, soil nutrient and moisture status, biome type and the successional stage of a biome. NPP determines the Net Ecosystem Productivity (NEP) in an area, together with the heterotrophic soil respiration. NEP represents the net C flux between the atmosphere and terrestrial ecosystems. Soil respiration depends on the C stocks in the different soil compartments (i.e. litter, humus and charcoal), their turnover rates and environmental conditions (i.e. soil water availability and temperature). All fluxes are calculated on a monthly basis, while the carbon pools are updated annually.

### Model application and experimental design

The IMAGE 2 model, along with the methodology presented here, has been applied to a number of experiments to show different sequestration potentials of C plantations up to 2100 under different baseline scenarios and management options. The experiments form variants to the implementation of the IPCC SRES A1b and B2 baseline scenarios [20,59]. The two baseline scenarios differ considerably in socio-economic and population developments (Table 8). In the B2 scenario, the demands up to 2050 for goods (e.g. food, timber and biofuels) are lower than in A1b baseline. But between 2050 and 2100, the demands remain high in B2, and drop in the A1b scenario. Combined with lower yield increases in the B2 world due to lower economic development and a fragmented world (e.g. leading to less technology exchange), less agricultural land is projected as being available for C plantations in the B2 scenario than in the A1b scenario. The consequences for the atmospheric CO_2 _concentration and global climate in the two scenarios are given in Table 8. Regionally, large temperature changes (up to 6°C) are simulated for the high latitudes, the Amazonian region, southern Africa and India.

**Table 8:** Main global characteristics of the IPCC A1b and B2 baseline scenarios (derived from The IMAGE Team [59]).

**Variable**	**Year**	**A1b**	**B2**
Population	2020	7.6	7.7
(10^9 ^people)	2050	8.7	9.4
(in 2000: 6.1)	2100	7.1	10.4

GDP/capita	2020	8.8	7.6
(10^3 ^US $ yr^-1^)	2050	24.2	13.7
(in 2000: 5.3)	2100	86.2	27.7

Extent arable land	2020	51.7	53.1
(Mkm^2^)	2050	53.1	53.6
(in 2000: 48.5)	2100	48.4	51.0

Atmospheric CO_2_	2020	426	421
Concentration (ppm)	2050	561	506
(in 2000: 375)	2100	753	606

Air temperature change (°C) (in 2000: 0.6)	2020	1.0	1.0
	2050	2.0	1.9
	2100	3.4	2.9

In the first set of experiments, the *physical sequestration potential *is estimated by establishing plantations wherever the carbon sequestration is higher than in the baseline (Table 9), with the exception of areas used for agriculture. The variants deal with permanent plantations in the A1b scenario (Exp.1) and frequently harvested plantations in the A1b (Exp. 2) and B2 (Exp.3) baseline scenarios. In the second set of experiments we assess the *social sequestration potential *by taking into account such barriers as no interference with the food supply and nature concerns. We implemented these criteria by establishing plantations on abandoned agricultural land only. Reforestation of harvested timberland is, for example, excluded, but could easily be incorporated in the methodology presented. Just as for the first set of experiments, we distinguish different types of management (Experiments 4 and 5) and baseline scenarios (Experiments. 4 and 6). In this set of experiments we assume that the plantations will actually be established, allowing for an evaluation of the possible role of carbon plantations in mitigating the build-up of CO_2 _in the atmosphere (Table 4).

**Table 9:** Overview of simulation experiments for the IPCC A1b or B2 baseline scenarios

	Plantation management	IPCC A1b	IPCC B2
Physical potential	Permanent	Experiment 1	
	Frequent harvest	Experiment. 2	Experiment 3
Social potential	Permanent	Experiment 4	
	Frequent harvest	Experiment 5	Experiment 6

## List of abbreviations

CO2: Carbon dioxide; Ha: Hectare; NPP: Net Primary Production; NEP: Net Ecosystem Productivity.

## Competing interests

The authors declare that they have no competing interests.

## Authors' contributions

The methodology and its applications were designed by JvM, who also prepared this manuscript. B.S. co-designed the methodology, implemented it in IMAGE 2 and contributed to the paper. BE contributed to the different stages of the methodology and earlier versions of this paper. RS and RL contributed to all stages of the paper. All authors read and approved the final manuscript.
